# Bartholin Gland Carcinoma: A State-of-the-Art Review of Epidemiology, Histopathology, Molecular Testing, and Clinical Management

**DOI:** 10.3390/cancers17233819

**Published:** 2025-11-28

**Authors:** Stoyan Kostov, Yavor Kornovski, Vesela Ivanova, Dimitar Metodiev, Angel Yordanov, Stanislav Slavchev, Yonka Ivanova, Anke Seidel, Ingolf Juhasz-Böss, Ihsan Hasan, Ibrahim Alkatout, Rafał Watrowski

**Affiliations:** 1 Research Institute, Medical University Pleven, 5800 Pleven, Bulgaria; drstoqn.kostov@gmail.com; 2 Department of Gynecology, Hospital “Saint Anna”, Medical University “Prof. Dr. Paraskev Stoyanov”, 9002 Varna, Bulgaria; ykornovski@abv.bg (Y.K.); st_slavchev@abv.bg (S.S.); yonka.ivanova@abv.bg (Y.I.); 3 Department of General and Clinical Pathology, Faculty of Medicine, Medical University Sofia, 1432 Sofia, Bulgaria; vivanova@medfac.mu-sofia.bg; 4 Neuropathological Laboratory, University Hospital “Saint Ivan Rilski”, 1431 Sofia, Bulgaria; dimitarmetodievvv@gmail.com; 5 Clinical Pathology Laboratory, MHAT “Nadezda” Women’s Health Hospital, 1373 Sofia, Bulgaria; 6 Department of Gynecologic Oncology, Medical University Pleven, 5800 Pleven, Bulgaria; angel.jordanov@gmail.com; 7 Institute of Pathology, Kartäuserstraße 51a, 79102 Freiburg, Germany; anke.seidel@ifpss.de; 8 Department of Obstetrics and Gynecology, Medical Center-University Hospital Freiburg, 79106 Freiburg, Germany; ingolf.juhasz-boess@uniklinik-freiburg.de; 9 University Specialized Hospital for Active Treatment in Oncology “Prof. Ivan Chernozemski”, 1756 Sofia, Bulgaria; ihsan_hasanov@abv.bg; 10 Department of Obstetrics and Gynecology, University Hospitals Schleswig-Holstein, Campus Kiel, 24105 Kiel, Germany; ibrahim.alkatout@uksh.de; 11 Department of Obstetrics and Gynecology, Helios Hospital Müllheim, 79379 Müllheim, Germany; 12 Faculty of Medicine, University of Freiburg, 79106 Freiburg, Germany

**Keywords:** Bartholin gland carcinoma, vulvar cancer, major vestibular glands, squamous cell carcinoma, adenocarcinoma, adenoid cystic carcinoma, human papillomavirus (HPV), HPV-associated tumors, rare gynecologic tumor, molecular profiling

## Abstract

Bartholin gland carcinoma (BGC) is a rare malignancy, comprising 3–7% of vulvar cancers and <1% of gynecologic tumors. Due to its low incidence, high-level evidence is lacking, and management is largely extrapolated from vulvar cancer guidelines. This comprehensive narrative review synthesizes current evidence on BGC, emphasizing histotype-specific features, diagnostic criteria, molecular profiling, and treatment strategies. The three most common subtypes are squamous cell carcinoma, adenoid cystic carcinoma (AdCC), and adenocarcinoma. HPV-associated tumors tend to occur in younger women and carry favorable prognoses. Accurate diagnosis requires exclusion of metastases and integration of clinical, imaging, and immunohistochemical data, including p16/HPV for squamous tumors, MYB/MYBL1 fusions for AdCC, and CK20/CDX2/SATB2 for intestinal-type adenocarcinoma. Approximately 50% of cases are diagnosed at an advanced stage due to misclassification as benign cysts or abscesses. Nodal metastasis occurs in >40% of cases, with histotype influencing prognosis. Adenocarcinoma and node-positive disease independently predict worse survival. Treatment hinges on complete surgical excision with 2–3 mm margins, bilateral groin evaluation, and histology-tailored adjuvant therapy. Emerging data support the use of immune checkpoint inhibitors in squamous BGC and targeted agents (e.g., mTOR/CDK4/6 inhibitors) in adenocarcinoma. We propose a practical molecular testing algorithm and highlight the urgent need for prospective, multinational collaboration to establish BGC-specific guidelines.

## Simple Summary

Bartholin gland carcinoma is a rare type of vulvar cancer that is often mistaken for a harmless cyst or infection, especially in women over 40. This can delay diagnosis and treatment. There are three main types—squamous cell, adenoid cystic, and adenocarcinoma—each behaving differently and needing specific tests to confirm. Some tumor types are linked to HPV, while others are not and may behave more aggressively. Due to its rarity, treatment is often guided by general vulvar cancer recommendations. Surgery is the mainstay, sometimes followed by radiation or chemotherapy. Advances in molecular medicine can improve diagnosis and guide newer treatments, like immunotherapy or targeted drugs. This review brings together all current knowledge about diagnosis and treatment of this rare cancer.

## 1. Introduction

Bartholin gland carcinoma (BGC) is uncommon, accounting for 3–7% of vulvar malignancies and <1% of gynecologic cancers [1,2]. It most commonly affects women aged 50–60 years, with a median diagnosis age of 53 years [1,3,4]. BGC is often diagnosed at a younger age than other vulvar malignancies [4,5]. Tumors typically arise in the posterolateral vestibule (4 and 8 o’clock), with frequent bilateral inguinofemoral drainage. Approximately 50% of cases are identified at an advanced stage, frequently due to initial misdiagnosis as benign Bartholin gland (BG) cysts or abscesses [6,7], leading to delays in appropriate treatment.

Primary BGC is best defined by tumors arising in BG tissue, supported by (1) location compatible with the gland; (2) histologic transition from non-neoplastic BG duct/acini to tumor where present, and (3) exclusion of another primary site. Histologically, squamous cell carcinoma (often HPV-associated) is most common at 30.7%, followed closely by adenoid cystic carcinoma (AdCC) at 29.6% and adenocarcinoma at 25% [3]. AdCC shows perineural tropism and distinct behavior, while adenocarcinoma—including intestinal type—must be differentiated from metastatic lower-GI primaries [1,6].

Because prospective trials are lacking, recommendations largely extrapolate from vulvar cancer guidelines. This narrative review synthesizes current evidence on diagnosis and management of BGC, compares major society guidance, and provides histotype-specific testing and treatment considerations, including a practical molecular panel and targeted-therapy opportunities.

## 2. Materials and Methods

### 2.1. Design and Data Synthesis

We performed a structured narrative review, with thematic synthesis organized by epidemiology/diagnosis, histotype-specific pathology and molecular testing, nodal management, primary surgery and margins, adjuvant therapy, recurrence/advanced disease, and follow-up. Given clinical and methodological heterogeneity and the rarity of BGC, quantitative pooling was not attempted. Where evidence was extrapolated from vulvar cancer more broadly, this is explicitly indicated.

### 2.2. Sources and Search Strategy

A comprehensive literature search was performed from database inception to August 2025. We searched MEDLINE (via PubMed), EBSCO (including 37 databases), and BASE (Bielefeld Academic Search Engine). Google Scholar and reference lists of recent reviews were used to identify additional records and grey literature. A representative core search string was: *((*“*Bartholin gland*” *OR* “*Bartholin’s gland*”*) AND (carcinoma OR cancer OR* “*adenoid cystic*” *OR adenocarcinoma OR squamous OR* “*squamous cell*” *OR neoplasm OR tumor*)), which was supplemented by targeted ancillary searches using combinations of diagnostic, anatomical, molecular, and treatment-related terms. No language restrictions were applied.

### 2.3. Eligibility Criteria

We included research papers and case reports reporting on primary BGC (any histotype) that provided clinical, pathological, treatment, or outcome data, guideline documents, and evidence syntheses (systematic reviews, meta-analyses). Editorials without data and conference abstracts without a full report were excluded. Studies describing secondary involvement of the Bartholin region by non-BG primaries were included only if directly informative for differential diagnosis.

### 2.4. Study Selection and Data Extraction

Titles/abstracts were screened, and potentially relevant full texts were reviewed. Two authors (S.K. and R.W.) were responsible for the final text corpus, after discussion and consensus. Extracted variables included study design, sample size, histotype, stage, nodal status, surgical margins and procedures, use of sentinel lymph node biopsy vs. inguinofemoral lymphadenectomy, adjuvant therapy, recurrence patterns, survival outcomes, and reported molecular alterations/biomarkers.

The most recent versions of major guidelines (e.g., NCCN v3.2024, ESGO 2023, BGCS 2023/24) were reviewed in full. We extracted explicit statements on indications for sentinel lymph node biopsy, inguinofemoral dissection, margin management, indications/fields for adjuvant radiotherapy or chemoradiation, and follow-up schedules. Differences and commonalities are summarized in a comparison table.

### 2.5. Quality Considerations

The credibility and relevance of included papers were assessed according to the Scale for the Assessment of Narrative Review Articles (SANRA), which constitutes an established appraisal tool for the assessment of non-systematic reviews [8]. It comprises six aspects, which guided prioritization of works selected for narrative synthesis: topic importance (item 1), statement of the aims (item 2), description of the literature search (item 3), referencing (item 4), scientific reasoning (item 5), and presentation of relevant and appropriate endpoint data (item 6) [8]. Where appropriate (management recommendations), we qualitatively appraised study design and sample size using established levels of evidence as proposed by the Oxford Centre for Evidence-Based Medicine [9].

### 2.6. Aims

Our aims were to (1) provide a detailed anatomical description with functional and surgical implications, (2) summarize epidemiology, clinical presentation, pathology, and outcomes of primary BGC, (3) describe histotype-specific features (squamous cell carcinoma, adenoid cystic carcinoma, adenocarcinoma), including molecular characteristics and their relevance for testing, and (4) translate direct and extrapolated evidence into management recommendations.

## 3. Anatomy of the Bartholin Glands

### 3.1. Morphology

The BGs’ anatomical position was first described in 1677 by the Danish anatomist Caspar Bartholin the Younger [10]. The BGs (major vestibular glands) are paired, pea-sized, mucin-secreting glands that provide vulvar and vaginal lubrication [10,11,12]. They are analogous to the male bulbourethral (Cowper’s) glands. The BG is typically ovoid (average size 0.5 cm) and usually not palpable; it becomes active after menarche. The glands lie in the posterior part of the vestibule at approximately the 4 and 8 o’clock positions [10,13], deep to the vestibular mucosa and inferior/lateral to the bulbocavernosus in the superficial perineal compartment [14]. Each gland gives rise to a single excretory duct (approximately 2–2.5 cm in length), which runs antero-supero-medially and opens near the vaginal introitus; ostial position and duct length show some variability across sources (e.g., 4/8 to 5/7 o’clock) [10,11,13]. A urogenital sinus origin is widely cited by analogy to male bulbourethral glands; however, modern lineage-tracing evidence is limited and such statements should be regarded as plausible rather than proven [10].

The blood supply is commonly attributed to branches of the external pudendal artery, and pudendal nerve innervation is frequently cited, but direct gland-specific angiographic or histologic mapping is limited; consequently, these are best considered the current best available descriptions rather than definitive [10,11,15].

With respect to lymphatic drainage, most authors report drainage to superficial and deep inguinofemoral nodes, but BG-specific mapping is sparse and underreported [11,15]. A recent anatomical study in 10 female cadavers suggested heterogeneous pathways, including a superficial inguinal route (most common), an internal pudendal pathway (through the pararectal fossa), and pelvic routes (including a labiocrural track), with predominantly ipsilateral flow; these findings may explain occasional atypical clinical patterns [16]. Historical and contemporary reports sometimes conflate major with minor vestibular glands; measurements for size, duct length, and ostial position in secondary sources may therefore vary. In this review, “BG” refers to major vestibular glands unless specified [10].

### 3.2. Histology

The vestibular orifice of the BG duct is composed of squamous epithelium. The main duct near the vestibular orifice is also lined by squamous epithelium, transitioning to a transitional/urothelial-like lining where the duct reaches the gland; secondary ducts are thinner and transitional. Single neuroendocrine cells are observed in the main duct. The main part of the gland is composed of high columnar epithelium; mucin-secreting columnar epithelial cells, myoepithelial cells, and neuroendocrine cells are the principal elements [2,3,10,14]. The myoepithelial cells form the basal cell layer of the glandular epithelium and may undergo multidirectional differentiation [2,3,10,14]. Abundant intracellular and luminal mucin underscores the lubricative secretory role [10]. Prostatic-type immunophenotypes (e.g., NKX3.1) have been described in major/minor vestibular glands and may confound surgical pathology differentials; this should be considered in BGC work-ups [10,17].

### 3.3. Function

The BGs contribute to introital lubrication, consistent with their mucin-rich acini and ductal architecture. Limited evidence suggests a role for neuroendocrine signaling and stimulus–secretion coupling, but proposed links to orgasm remain unproven in humans [10,11,12]. Nerve-fibre markers in vestibular mucosa adjacent to ducts (e.g., PGP 9.5, CGRP, S-100; parasympathetic/sympathetic markers) indicate nearby autonomic and nociceptive fibres; quantitative work also shows increased PGP 9.5-positive innervation in the vestibule in vulvodynia, supporting a dense nociceptive network in this region [10,18,19,20]. Whether these fibres specifically enter the major gland is unresolved.

## 4. Bartholin Gland Carcinoma

### 4.1. Definition

The first diagnostic criteria for BGC were described by Honan in 1897: (1) anatomical location of the tumor in the BG area, (2) intact overlying skin, (3) the deep portion of the tumor within the labia majora, (4) presence of residual normal glandular elements, (5) histology consistent with BG origin, and (6) no evidence of a concurrent primary tumor elsewhere [3,21]. These were replaced by the Chamlian and Taylor criteria in 1972 because the Honan set is too restrictive, particularly in advanced disease where overlying skin or normal glandular elements may be entirely replaced by tumor. The revised criteria are as follows: (1) areas of apparent transition from normal BG tissue to malignant elements on histological examination, (2) a tumor located in the BG area and histologically consistent with BG origin, and (3) no evidence of a concurrent primary tumor elsewhere (i.e., metastasis to the BG excluded) [3,22].

In many cases, these criteria cannot be fulfilled because adjacent normal BG tissue is wholly replaced by tumor [23]. However, this point remains debated. For example, Cardosi et al. [6] included women as BGC even when the tumor completely replaced the BG and no benign-malignant transition was demonstrable, provided there was no overlying skin involvement or ulceration.

### 4.2. Epidemiology

The incidence of BGC is higher among postmenopausal women; however, compared with other vulvar cancers, the age at diagnosis tends to be younger. Cases in young women have been reported [24,25,26,27]. In a single-center U.S. series, BGC was reported more often in African American women, but this finding may reflect local demographics or referral patterns rather than a true biological predisposition and may not be generalizable [5]. Patients with BGC are more often diagnosed at an advanced stage than those with other vulvar cancers [5]. Human papillomavirus (HPV) infection is mainly associated with squamous histology, with HPV16 as the most observed subtype [28]. Occurrence of BGC does not appear related to prior procedures in the BG region (e.g., marsupialisation, incision, episiotomy). In a systematic review of primary carcinomas at episiotomy scars, Palicelli et al. [29] found only one case of AdCC of the BG, which was probably incidental rather than causally linked to the episiotomy. BGC has been reported more often in the left gland than the right [30].

### 4.3. Clinical Manifestations

Symptoms are non-specific, and approximately 40% of patients have no signs or symptoms. The leading symptom is a painful vulvar mass in the BG area; other commonly observed symptoms include bleeding, pruritus, skin discoloration, and dyspareunia [3,5]. Perineural invasion (typical for AdCC) can produce a burning sensation even before a palpable mass is detected [4]. Pain in the inguinofemoral region may indicate fixed metastatic lymph nodes [2,3].

### 4.4. Diagnosis

The correct diagnosis of BGC is delayed in about 50% of cases, as lesions are often presumed to be abscesses or cysts. Given the high incidence of BG abscesses/cysts and the rarity of BGC, general recommendations for biopsy remain cautious. In our practice, we routinely perform an ultrasound for each patient presenting with an enlarged BG. This approach is quick, informative, and supported by the literature [31,32]. Because diagnostic delay is common and incidence increases with age, biopsy should be performed in patients with a persistent or progressive BG mass, solid masses or solid components within a presumed “cystic lesion,” or lesions invading surrounding tissues, and should be generously considered in peri- and postmenopausal patients [3,4,13,32,33,34]. Formal en bloc excision is often considered superior to a limited incisional biopsy when feasible, as small biopsies may be insufficient to meet diagnostic criteria and carry a risk of cyst/gland capsule rupture [4]. If an incisional biopsy is performed, it should include the lesion edge to capture transition from normal to carcinomatous tissue and be deep enough to assess depth of invasion/stage [35].

The vulvar-anal region and vagina should be carefully inspected. The uterine cervix should be evaluated with a Pap smear and HPV testing; colposcopy with biopsy is indicated for an abnormal Pap or a positive HPV test. Regional inguinofemoral nodes should always be palpated; nodal status can be further assessed with ultrasound-guided sampling (core-needle biopsy or fine-needle aspiration) [36,37]. If pain is significant, the pelvic examination may be conducted under general anesthesia and can be combined with cystoscopy and/or proctoscopy/colonoscopy depending on disease extent. Tumor size and infiltration into underlying tissues should be assessed. A complete gynecologic examination including uterus, ovaries, and breasts should be performed, as BG metastases from ovarian, endometrial, and breast cancers have been occasionally reported [38,39,40,41], and synchronous ovarian and BG cancers have been described [42]. 

Imaging modalities include expert vulvar ultrasonography, computed tomography (CT), magnetic resonance imaging (MRI), and positron emission tomography–computed tomography (PET-CT). CT should assess the chest, abdomen, and pelvis including the vulva and inguinofemoral regions, whereas MRI is more suitable to verify brain metastases. Both brain and pulmonary metastases from BGC have been reported [43,44]. T2-weighted MRI is preferred to define tumor dimensions and local invasion of adjacent structures (anterior rectal wall, posterior bladder wall, urethra) and to distinguish recurrence from post-surgical or post-radiotherapy changes [2,3,4]. PET-CT is mainly used to evaluate distant metastases and regional/distant lymph-node status. Although PET-CT may detect small nodal metastases, no imaging modality provides both high sensitivity and specificity for fully staging disease or detecting deep pelvic nodal metastases [2,3].

Table A1 summarizes preoperative diagnostic modalities in BGC (with VSCC comparators) and indicates the evidence source (direct vs. indirect) and OCEBM level [9]. The molecular diagnostic work-up and the differential diagnosis of BGC are discussed in subsequent sections of this review.

### 4.5. Staging

The FIGO staging system for vulvar cancer, last revised in 2021 by the FIGO Committee on Gynaecologic Oncology, applies to BGC [45].

### 4.6. Differential Diagnosis

BGC is most commonly misdiagnosed as a BG cyst or abscess. Less common differential diagnoses reported in the literature include nodular hyperplasia, adenoma and adenomyoma; leiomyoma and leiomyosarcoma; endometriosis and endometrioma; lymphoma; arteriovenous malformation; hamartoma; papilloma; malignant melanoma; malakoplakia; tuberculosis; and non–BG masses arising in or adjacent to the vaginal vestibule [32,46,47,48,49,50,51,52,53,54,55].

## 5. Histological BGC Subtypes

Histological carcinoma subtypes reported in the BG include squamous cell carcinoma (SCC), adenocarcinoma, AdCC, transitional (urothelial-type) carcinoma, neuroendocrine carcinoma, undifferentiated carcinoma, adenosquamous carcinoma, and epithelial-myoepithelial carcinoma.

### 5.1. Histological Distribution

The largest systematic analysis [3] of 275 malignant BG tumors with known histotype (including sarcomas and other rare subtypes) reported the following distribution:

- SCC: 80 cases (30.7%);
- AdCC: 77 cases (29.6%);
- Adenocarcinoma: 65 cases (25%);
- Transitional cell carcinoma: 7 cases (2.6%);
- Sarcoma: 7 cases (2.6%);
- Other rare subtypes: 38 cases (14.6%)

### 5.2. Squamous Cell Carcinoma

Squamous cell BG carcinoma (SCC-BG) is the most common histological subtype of BGC. Its incidence among malignant epithelial tumors of the gland ranges from 31% to 88% [5]. As in vulvar cancer, SCC-BG includes keratinizing and non-keratinizing forms. SCC-BG arises from the duct or vestibular orifice of the gland and typically infiltrates adjacent tissue; late ulceration after tumors become large is common [14]. Risk factors and histopathology mirror vulvar SCC; accordingly, SCC-BGs are categorized as HPV-associated or HPV-independent. An association between HPV and BG SCC has been reported in multiple studies [28,56,57].

HPV-associated SCC-BG appears more frequent than HPV-associated vulvar SCC (where HPV-independent disease is at least as common). Patients with HPV-associated BGC are often younger and may have a history of cervical intraepithelial neoplasia, consistent with lower-genital-tract HPV infection [56]. In one series of 12 BG SCCs, all were HPV-associated [57]. Similarly, among seven BGC cases, HPV-16 was detected in six; the HPV-negative case was an adenocarcinoma [28]. HPV-16 is the most common genotype in squamous BGC [28].

p16 over-expression correlates with transcriptionally active high-risk HPV in vulvar, cervical, and BG cancers. In selected cases, p16 immunohistochemistry (IHC) may be positive despite HPV PCR negativity, consistent with the “hit-and-run” hypothesis. After oncoprotein (E6/E7)–driven initiation and heritable reprogramming, viral genomes may be lost yet tumorigenesis persists [58,59]. HPV-associated squamous BGC is linked to better prognosis, lower FIGO stage, negative inguinofemoral nodes, and younger age at diagnosis [57,60]

Rare squamous subtypes include basaloid and lymphoepithelioma-like carcinomas, both associated with HPV, similar to cervical cancer [61,62].

**Figure 1:**
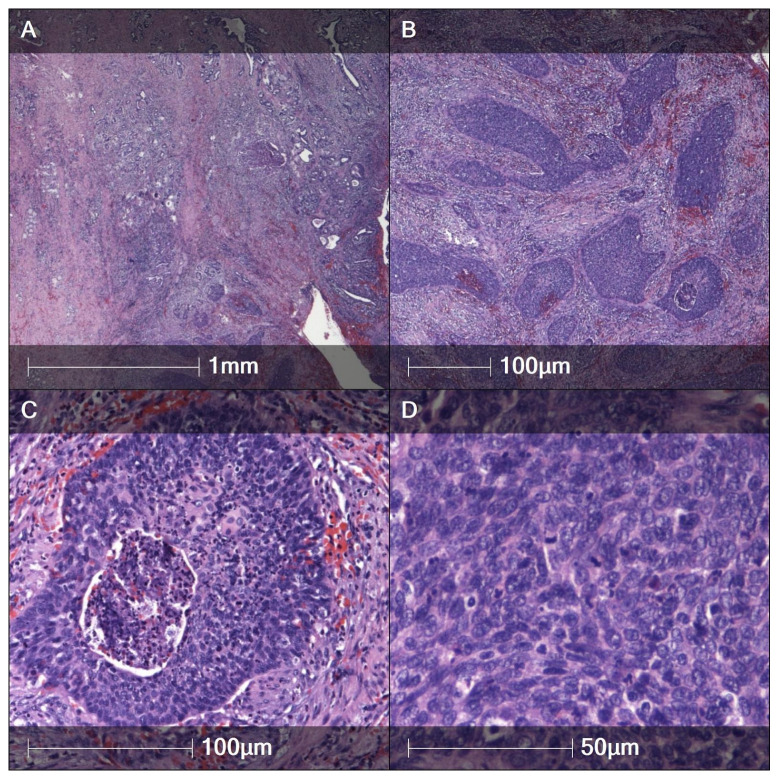
SCC-BG. (**A**) Low-power overview with infiltrative nests in desmoplastic stroma adjacent to Bartholin duct/acini. H&E, ×20. (**B**) Irregular anastomosing nests and cords with brisk stromal reaction. H&E, ×100. (**C**) Tumor nest with central comedo-type necrosis (arrow). H&E, ×200. (D) Marked nuclear pleomorphism with atypical mitotic figures; keratinization scant. H&E, ×400.

**Figure 2:**
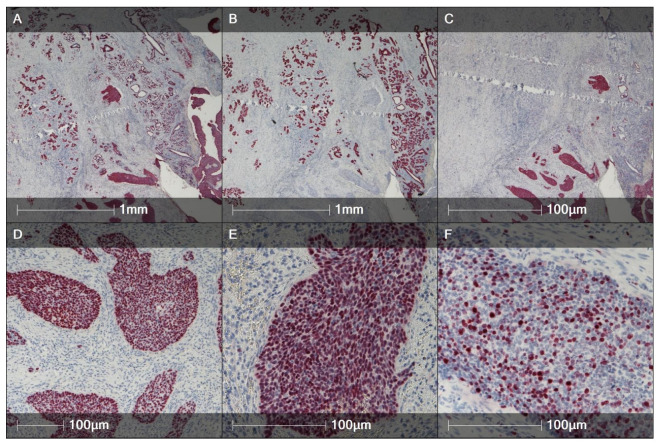
SCC-BG. (**A**) Diffuse CK5 cytoplasmic/membranous immunopositivity in invasive nests. Hematoxylin counterstain, ×20. (**B**) CK7 highlights residual Bartholin duct/acini epithelium; tumor nests largely negative. Hematoxylin counterstain, ×20. (**C**) p16 shows diffuse block-type nuclear and cytoplasmic positivity in tumor nests, consistent with HPV-associated SCC. Hematoxylin counterstain, ×20. (**D**) p40: diffuse strong nuclear staining in tumor cells. Hematoxylin counterstain, ×100. (**E**) p63: diffuse nuclear positivity supporting squamous/basal phenotype. Hematoxylin counterstain, 200. (**F**) Ki-67: high labeling index within tumor nests. Hematoxylin counterstain, ×200.

A histological picture of HPV-associated BG-SCC with characteristic immunohistochemical features is presented in Figure 1 and Figure 2.

### 5.3. Adenocarcinoma

Adenocarcinomas of the BG may arise from tubules, acini, or the ductal epithelium [14]. Most are mucin-producing, with patterns ranging from papillary to mucoepidermoid or mucinous [63]. Tumor cells often contain intracytoplasmic mucin and may show papillary architecture and CEA positivity. These tumors tend to infiltrate deep perineal tissues along nerves, with frequent ischioanal fossa involvement. Cystic changes with mucinous contents are common [14]. Ulceration and cutaneous involvement are less frequent than in SCC [57].

Many authors describe SCC and adenocarcinoma as the two most common BGC subtypes (together 80–90%, with similar frequencies) [2,21]. However, a systematic review found adenocarcinoma to be the third most common histology after SCC and AdCC (ca.25%) [3]. Unlike cervical adenocarcinoma, BG adenocarcinoma is generally HPV-negative [57,63,64] and may be more aggressive, with a higher incidence of regional nodal metastasis than SCC-BG [5,7,57].

Immunophenotypes can overlap with intestinal differentiation. ER/PR, GCDFP-15, mammaglobin, and GATA3 are often negative, whereas CK7, CK20, CDX2, CEA, and CK19 may be positive. KRAS mutations have been reported, and BG intestinal-type adenocarcinoma shares features with colorectal adenocarcinoma [1]. KRAS variants were identified in one of two reported intestinal-type mucinous BGCs [65,66]. Mammary-like adenocarcinomas of the vulva can mimic BG adenocarcinoma and should be considered in the differential [57]. 

**Figure 3:**
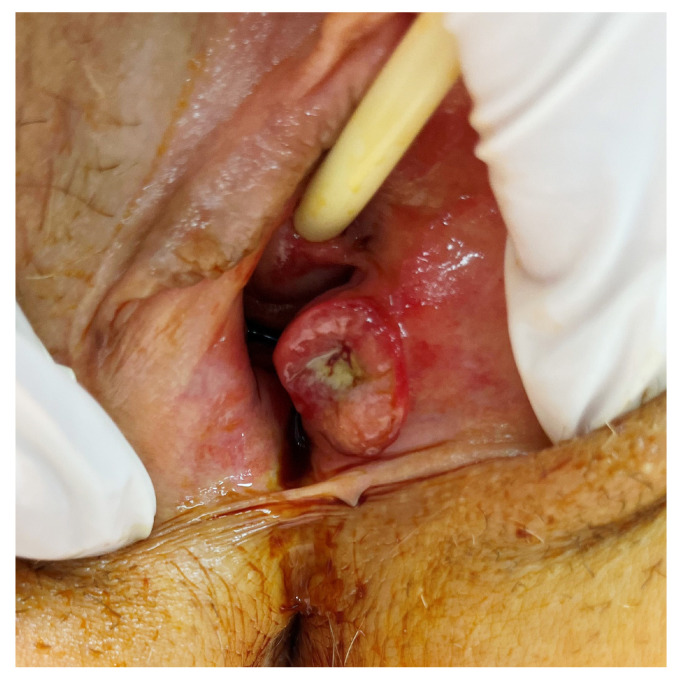
Gross appearance of an adenocarcinoma of the left BG (reproduced with patient’s consent).

**Figure 4:**
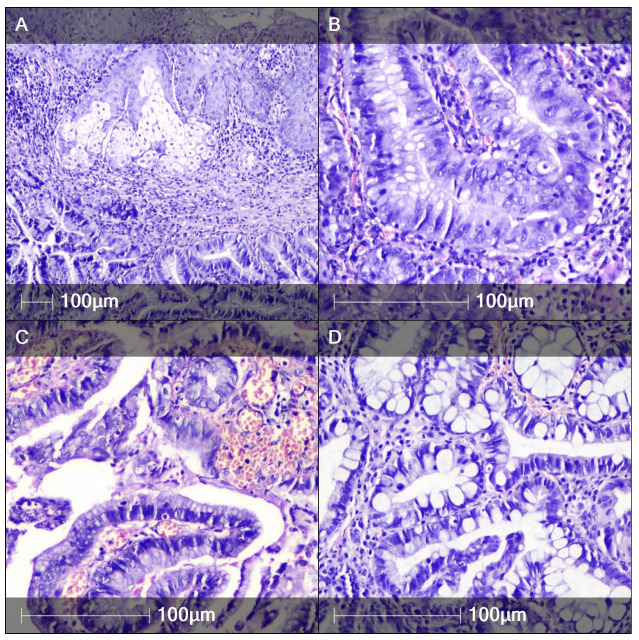
Adenocarcinoma of the BG. (**A**) Focus on atypical glandular structures in close proximity to overlying squamous epithelium and sebaceous gland unit. H&E, ×100. (**B**) Closer view of atypical gland with marked nuclear pleomorphism and presence of mitotic figures. H&E, ×200. (**C**) Thick core papillary architectural pattern of growth. H&E, ×200. (**D**) Intestinal differentiation within the neoplastic epithelium. H&E, ×200.

Rare primary clear cell adenocarcinoma of the BG has also been reported and should be considered in the differential [65,66,67,68]. Macroscopic and histologic features of BG adenocarcinoma are shown in Figure 3 and Figure 4.

### 5.4. Intestinal-Type (Cloacogenic) Adenocarcinoma

Intestinal-type (cloacogenic) adenocarcinoma of the BG is rare; fewer than 30 vulvar intestinal-type cases have been reported [68]. Two main hypotheses exist, i.e., (1) malignant transformation of embryonic cloacal remnants (“neometaplasia”) [68,69]; and (2) intestinal metaplasia or ectopic intestinal epithelium within Müllerian-derived tissues. Metastasis from colorectal mucinous adenocarcinoma must be excluded, and KRAS testing can aid classification [66,68]. A recent BG case with literature review documented CK7−/CK20+/CDX2+ intestinal-type mucinous adenocarcinoma, negative for GATA3, mammaglobin, and GCDFP-15, p16-negative, MMR-proficient, with a KRAS exon 2 p.G12D mutation (colonoscopy and cross-sectional imaging excluded a colorectal primary) [70]. These findings support a directed immunopanel (CK7/CK20/CDX2 ± p16 and breast markers), colonoscopy/imaging, and KRAS testing when intestinal-type morphology is present [70]. Inguinofemoral nodal metastasis occurs in ca. 20% of vulvar intestinal-type adenocarcinomas [68].

### 5.5. Adenoid Cystic Carcinoma

AdCC of the BG resembles AdCCs of salivary, lacrimal, and other exocrine glands [1]. One review reported an incidence of ca.29.6% among BGCs [3], though many series estimate ca.10–15% [35,71]. AdCC likely arises from myoepithelial cells [72]. Histologically, AdCC shows solid, tubular, and cribriform patterns; grading is based on the proportion of solid growth: grade 1 (no solid areas), grade 2 (<30% solid), grade 3 (≥30% solid) [73]. A higher solid component portends worse outcomes. Cribriform tumors exhibit cords/nests of uniform small cells with cribriform architecture and acellular spaces containing mucin or hyalinized material [3,73,74]. High-grade transformation of BG AdCC has been reported and confers a poorer prognosis [75].

Immunohistochemically, tumor cells often express carcinoembryonic antigen, keratins, lysozyme, S-100 protein, and lactoferrin [14]. Additional markers include smooth-muscle myosin, KIT/CD117, SMA, SMM, S-100, CD43, CEA, vimentin, and MYB [2,3,13]. The median age at diagnosis is circa 59 years [76]. AdCC is characterized by slow growth, local invasion, and a high rate of locoregional recurrence driven by perineural invasion—a hallmark of this histotype [77]. Local recurrences can occur despite negative margins due to early perineural spread; burning dysesthesia (± pruritus/paresthesia) may precede detection of a mass and often intensifies with tumor growth [2,14]. BG AdCCs are typically HPV-negative in contrast to some sinonasal or cervical AdCCs [78].

Regional nodal metastases are less common than in other BGC histotypes and, when present, tend to be ipsilateral. In contrast, late distant metastases are relatively frequent, particularly to the lungs or brain [35,44,71,76]. Reported rates of local recurrence and distant metastasis are 30% and 31%, respectively [76,79], with local recurrence typically preceding distant spread [71]. An older series noted pregnancy in 50% of patients with BG AdCC; this observation has prompted speculation about pregnancy as a potential risk factor, but evidence remains limited [71,79].

### 5.6. Transitional Cell Carcinoma

Transitional (urothelial-type) carcinoma of the BG arises from the transitional epithelium lining the ducts. Fewer than 10 cases have been described. Tumors comprise malignant urothelial-type cells with CK7 and CK20 positivity; some cases are HPV-associated [80,81,82].

### 5.7. Epithelial-Myoepithelial Carcinoma

Epithelial—myoepithelial carcinoma (EMC) of the BG is extremely rare and resembles its salivary-gland counterpart. As with BG AdCC, a pregnancy-associated vulvar EMC has been described [83]. Vulvar EMC appears slightly more common than BG EMC. Reported low-grade EMCs show favorable outcomes (no perineural/vascular invasion or distant metastasis), whereas many high-grade vulvar EMCs present with advanced locoregional disease and distant spread. Immunohistochemically, the inner epithelial layer stains for cytokeratin and EMA; the outer/myoepithelial layer is positive for p63, calponin, and α-smooth-muscle actin. Diffuse strong c-KIT immunoreactivity has also been reported [83,84,85]. Accurate diagnosis can be challenging and may require a broad immunopanel (and, rarely, electron microscopy) [3].

### 5.8. Neuroendocrine Carcinoma (Small-Cell Type)

Small cell neuroendocrine carcinoma of the BG is exceptionally rare and clinically aggressive, with a tendency to early nodal and distant spread; only a handful of primary cases are convincingly documented [24,86,87,88]. A plausible histogenetic source is the scattered neuroendocrine cell population described within BG ducts/acini [89]. Morphology mirrors pulmonary small cell carcinoma: sheets/nests of hyperchromatic small cells with scant cytoplasm, nuclear molding, brisk mitoses, and necrosis/apoptotic debris. Immunophenotype is that of a high-grade NEC: cytokeratins (e.g., CAM 5.2/AE1-AE3) with synaptophysin, chromogranin A, CD56 (often also NSE), and a very high Ki-67; CD10 may be positive in some cases [87,88]. Because metastatic small cell carcinoma (especially pulmonary) to the vulva is far more common than a true primary, thorough clinical and imaging work-up is essential; Merkel cell carcinoma has also been reported in the vulvar/peri-Bartholin region and should be separated from glandular SCNEC on clinicopathologic grounds [90].

### 5.9. Mixed Tumors

“Mixed” BG neoplasms are among the rarest BGCs, with fewer than five reported cases. A combined AdCC and SCC of the BG was described by Webb et al. [91]. Salivary-gland-type mixed tumors probably arising from the BG have included benign pleomorphic adenoma and carcinoma ex pleomorphic adenoma with adenoid cystic, glandular, and undifferentiated components set in a chondromyxoid matrix with cartilage/bone, mirroring salivary-gland counterparts [92]. A third report documents a mixed carcinoma with three-directional differentiation (predominantly papillary noninvasive transitional-cell pattern) occurring 28 years after pelvic radiotherapy for cervical cancer [93].

## 6. Molecular Profiles of BGC

The molecular profile of BGC mirrors its histology. Because dedicated next-generation sequencing (NGS) datasets for BGC remain scarce, we explicitly indicate where statements are extrapolated from larger vulvar SCC (VSCC) cohorts and keep such inferences conservative. Where direct BGC evidence exists, we prioritize it. This section integrates high-value diagnostic markers with pragmatic testing steps and clarifies which signals carry therapeutic implications. Molecular characteristics of BGC histotypes are provided in Table 1.

### 6.1. Squamous Cell BGC

SCC-BG is most often HPV-associated. In the largest single-institution BGC series, all evaluable SCC-BGs showed diffuse, strong p16 expression, and earlier work detected HPV16 DNA in most tested tumors [28,57]. It is therefore appropriate to confirm a transcriptionally active HPV pathway in SCC-BG with p16 IHC, supplemented where available by HPV RNA/DNA in situ hybridization (ISH). Recent VSCC cohorts support a three-tier molecular classification that integrates HPV and p53 status with prognosis—HPV-associated, HPV-independent/p53-wildtype, and HPV-independent/p53-abnormal—where HPV-associated tumors show the most favorable prognosis, HPV-independent/p53-abnormal the worst, and HPV-independent/p53-wildtype an intermediate outcome; this framework now underpins WHO 2020 terminology and ESGO/ICCR reporting recommendations [94,95,96,107]. The molecular profile data of SCC-BG are derived mainly from studies of the vulvar cancer profile, which is logical given shared risk factors and pathology; within this framework, HPV-related squamous tumors tend to harbor PI3K/mTOR-axis lesions, including alterations in PTEN, PIK3CA, SOX2, FBXW7 and STK11 (all based on VSCC). Whole-exome VSCC data further delineate recurrent alterations, including TP53 (67%), FAT1 (28%), CDKN2A (25%), RNF213 (23%), NFE2L2 (20%) and PIK3CA (20%), with CCND1 copy-number gains in 28% and universal MMR proficiency; TP53 mutation, CCND1 gain, and their combination are associated with poorer recurrence-free and disease-specific survival, and every tumor harbored at least one potentially actionable alteration [97,98]. By contrast, HPV-negative squamous BGC is expected to show frequent alterations in TP53 and CDKN2A, with HRAS mutations and amplifications of CCND1, EGFR and NOTCH1, as well as 9p24.1 (PD-L1/PD-L2) gains, again principally inferred from VSCC datasets [99,100,108]. Consistent with this biology, PD-L1 expression appears more common in HPV-negative than HPV-associated SCC (for example, approximately 33% vs. 9% in VSCC) [99,109]. In routine practice, pattern-based p53 IHC interpretation, recognizing aberrant patterns such as overexpression, null, or cytoplasmic staining can help flag an HPV-independent pathway in BGC, supporting triage to broader sequencing [96,107,110,111]. In HPV-independent disease, cyclin D1 overexpression (IHC ≥50% cells) functions as a surrogate for CCND1 gain (sensitivity 94%, specificity 67%) and independently predicts worse disease-specific survival; hence, in SCC-BG suspected to be HPV-independent, adding cyclin D1 IHC (and, when feasible, CCND1 copy-number by NGS/FISH) can refine prognosis and trial discussions [97,98]. Finally, VSCC studies describe an HPV-independent “HSIL-like” precursor that mimics HPV-associated HSIL morphologically yet is p16-negative/HPV-negative and often p53-abnormal, with higher recurrence risk; while not yet defined in BGC, this underscores the need for mandatory HPV/p16 testing when HSIL-like changes are encountered [95,96,111].

### 6.2. Adenocarcinoma

Adenocarcinoma immunophenotype must be interpreted alongside clinical and imaging data. Intestinal-type primaries usually express CK20 and CDX2 with variable CK7 and frequent SATB2 positivity, yet this profile overlaps with metastatic colorectal or anal-canal primaries; accordingly, a full gastrointestinal work-up is mandatory to exclude a secondary source [103,104,105]. Small recent series in the vulvar/vaginal setting report KRAS and TP53 variants with HPV-negative status. For non-intestinal BG adenocarcinoma, a whole-genome case revealed somatic PTEN loss (exons 2–5) together with CCND1 amplification; these findings supported everolimus (mTOR inhibitor) followed by palbociclib (CDK4/6 inhibitor) with radiologic responses under compassionate use [106].

### 6.3. Adenoid Cystic Carcinoma

AdCC of the BG (AdCC-BG) is characterized by activation of the MYB pathway. Canonical events include MYB::NFIB fusion or MYBL1 rearrangements with MYB protein over-expression; additional mutations are sparse and non-recurrent, with occasional single-case reports involving AKT1, KDM6A, GNAS, or GNAQ [101,102,112,113]. A somatic PLCG1 mutation has also been described in a woman with AdCC-BG [102]. AdCC-BG is considered not HPV-related, and p16 is not a reliable surrogate in this histotype [78]. The presence of MYB/MYBL1 alterations supports the diagnosis of AdCC-BG and helps distinguish it from non-AdCC, which generally lacks these rearrangements [78,101,114].

### 6.4. Testing Algorithm

**Figure 5:**
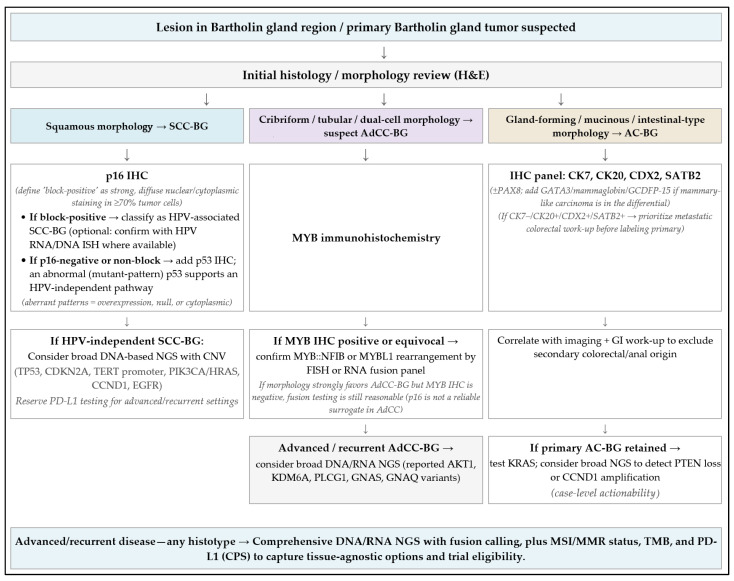
Histotype-oriented diagnostic testing algorithm for BGC.

Figure 5 illustrates a histotype-oriented diagnostic testing algorithm, and Table 2 summarizes potentially actionable biomarkers for BGC. For squamous lesions, routine p16 (± HPV ISH) and p53 patterning should be accompanied by a clear statement that the lesion is primary to Bartholin tissue (location, transition where present, exclusion of another primary). For adenocarcinomas, an intestinal-type immunophenotype (CK20/CDX2/SATB2) should prompt GI work-up to exclude metastasis. A practical diagnostic pitfall is NKX3.1 positivity in vestibular glands and lesions, which can simulate prostatic differentiation. This is important when evaluating unusual immunoprofiles or metastatic differentials [10,17]. Histologically, the duct–acinar transition (squamous → transitional/urothelial-like → mucinous columnar) and the presence of myoepithelial and scattered neuroendocrine cells set expectations for pathology reporting and margin assessment; they also explain mucin-rich tumor microenvironments in some histotypes [2,3,10,14]. 

In practice, ancillary testing should follow histotype. For SCC-BG, p16 IHC with confirmatory HPV RNA/DNA ISH where available, together with p53 IHC to identify HPV-independent biology that may warrant broader sequencing, is recommended. Where HPV-independent biology is suspected, adding cyclin D1 IHC and, when possible, CCND1 copy-number assessment can refine risk stratification [97,98]. For AdCC-BG, MYB protein IHC followed by targeted detection of MYB::NFIB or MYBL1 fusions using FISH or RNA-based assays is diagnostically high-yield. For adenocarcinoma, particularly intestinal type, an immunopanel including CK7, CK20, CDX2, and SATB2 (± PAX8) should be interpreted alongside a directed GI work-up to exclude metastasis. In any histotype with advanced or recurrent disease, broad DNA/RNA NGS with fusion calling, assessment of MMR/MSI and tumor mutational burden, and PD-L1 scoring can identify trial eligibility or tissue-agnostic options. Note that VSCC WES suggests MMR proficiency is typical, and signals for immune checkpoint inhibition in vulvar SCC derive largely from KEYNOTE-158 and related studies and are therefore extrapolated to BGC [115]. 

Given the rarity of BGC, most sequencing signals outside AdCC-BG derive from small series or are inferred from VSCC. We therefore emphasize diagnostic markers with immediate clinical utility (p16/HPV and p53 patterning in SCC; MYB/MYBL1 fusions in AdCC-BG; intestinal immunophenotype in adenocarcinoma) and recommend that extended genomic findings be discussed within a molecular tumor board.

## 7. Treatment

There is no established consensus or standard surgical treatment for BGC, due to the rarity of the disease. No level I evidence is available, as most studies are retrospective and include small cohorts. Table 3 consolidates evidence levels for major treatment modalities in BGC mapped to OCEBM categories [9] and indicates where recommendations are extrapolated from VSCC.

### 7.1. Surgical Treatment of the Primary Tumor

Surgical management generally follows principles used for medial vulvar cancer. For early-stage disease, procedures of varying radicality have been reported—radical local excision, simple hemivulvectomy, or radical vulvectomy. In one retrospective series comparing (1) radical local excision plus adjuvant radiotherapy, (2) radical vulvectomy plus adjuvant radiotherapy, and (3) radiotherapy alone, five-year disease-free survival was 86%, 78%, and 50%, respectively; less radical surgery followed by radiotherapy was associated with long-term survival and fewer complications [21]. The cornerstone remains complete excision; histologically clear margins are essential, with contemporary thresholds of approximately 1–2mm [123] or >2–3 mm [116] regarded as sufficient (earlier thresholds were ≥8 mm or 1 cm) [116,124].

#### Practical Surgical Considerations

Because the BGs sit deep to the vestibular mucosa at the 4/8-o’clock positions and abut the bulbocavernosus within the superficial perineal compartment, incision planning that respects the gland–duct axis helps balance margin clearance with preservation of introital function [11,13,14]. The arterial supply and specific glandular innervation are incompletely mapped and are often attributed to external pudendal branches and the pudendal nerve; in view of limited primary data, meticulous tissue handling and conservative assumptions about nerve pathways are prudent [10,11]. Pathology correlation should note that NKX3.1 expression in vestibular glands may mimic “prostatic-type” immunophenotypes in margins or small samples [10,17].

In AdCC of the BG, more radical procedures may be considered given the propensity for perineural invasion and higher risk of local recurrence [35]. For locally advanced tumors, two approaches are commonly used: primary resection followed by adjuvant radiotherapy/chemoradiation, or radical vulvectomy after neoadjuvant radiotherapy/chemoradiation. Ultraradical procedures (e.g., pelvic exenteration) may occasionally be required to obtain clear margins, particularly in adenoid cystic BGC with higher local recurrence rates, although neoadjuvant or definitive chemoradiation is often preferred because of morbidity and psychosexual impact [71,125]. In cases of anal involvement, neoadjuvant therapy followed by surgery may be considered. Limited urethral involvement can be addressed surgically if 1.5 cm of distal urethra is resected, a length generally not associated with postoperative incontinence [123,126]. Current guidelines for vulvar cancer recommend limiting radicality to preserve midline structures and function [35,116,123]. Re-excision is recommended when feasible for invasive disease with positive margins. No single reconstructive technique is preferred; reconstruction is undertaken when primary closure is challenging, to optimize cosmetic and functional outcomes [116].

### 7.2. Surgical Treatment of the Groin

In a systematic review, positive inguinofemoral nodes were reported in 41.1% (58/141) of cases with known nodal status; pelvic nodal metastases were identified in 7/54 with reported anatomic nodal positivity [3]. Given typical vestibular drainage, inguinofemoral evaluation is standard, while recognizing anatomic variability described in cadaveric mapping studies [10,11,16]. Groin dissection is generally indicated in all stages except T1a, and because BGC is typically medial, dissection is usually bilateral. Some authors question contralateral lymphadenectomy when ipsilateral nodes are negative because of morbidity. Dissection includes superficial inguinal and deep femoral nodes (with saphenous-vein preservation). Pelvic lymph-node dissection may be considered for bulky inguinofemoral disease and/or bulky pelvic nodes on imaging [2,3,35,116], although pelvic radiation is generally preferred. In a randomized trial of node-positive vulvar cancer, adjuvant groin/pelvic radiotherapy improved six-year overall survival compared with pelvic node dissection, particularly for bulky groin disease and in those with ≥2 positive nodes [127].

The value of groin dissection in AdCC of the BG remains uncertain, as lymph-node metastases occur in only 10% [35,117]. By contrast, adenocarcinoma and adenosquamous carcinoma show higher nodal involvement [64,118].

#### Sentinel Lymph-Node Biopsy (SLNB)

SLNB is recommended for tumors <4 cm or >T1a without suspicious nodes on examination/imaging [116]. Representative injection is not feasible when the tumor involves the vagina, anus, or urethra [35]. In BGC, SLNB is generally bilateral; if only an ipsilateral SLN is identified, contralateral inguinofemoral dissection is advised. Complete groin dissection is indicated when an ipsilateral SLN harbors macrometastasis (>2 mm). When SLNB is bilateral and only ipsilateral metastasis is present, the risk of contralateral metastasis is low and additional contralateral treatment may be omitted [116].

### 7.3. Radiotherapy Alone

Recommendations for adjuvant external-beam radiotherapy (EBRT) in BGC align with those for vulvar cancer [3,35,116,128,129].

#### 7.3.1. Adjuvant EBRT to the Vulvar Region

Adjuvant EBRT to the vulvar region is indicated in cases of positive postoperative margins when further surgical resection is not feasible, and clear but close postoperative margins (<2–3 mm) with perineural and/or lymphovascular space invasion and depth of invasion >5 mm.

#### 7.3.2. Adjuvant EBRT to the Groin

Adjuvant EBRT to the groin is indicated in following scenarios:

- Micrometastases after SLNB (<2 mm) or isolated tumor cells, as an alternative to groin dissection;
- Positive sentinel-node metastasis >2 mm and/or extracapsular spread, after completion dissection;
- As an alternative to groin dissection in the presence of bulky metastatic nodes;
- When contralateral inguinofemoral nodes are not dissected.

#### 7.3.3. Adjuvant EBRT to the Pelvis

Should be performed:

- When groin nodes are metastatic (field typically to the distal iliac chain up to the iliac bifurcation).
- When pelvic nodal metastases are suspected on imaging or pathologically proven (field one level above the highest involved node).

Bilateral pelvic radiation is indicated for bilateral groin metastases.

#### 7.3.4. Brachytherapy

Image-guided adaptive brachytherapy may be considered for positive margins and as dose escalation for residual tumor near the urethra or vagina; a high-dose-rate interstitial boost can be used in locally advanced BGC treated with primary chemoradiation [119]. 

#### 7.3.5. Practical Considerations

Target volumes are individualized by stage and patient factors. Adjuvant radiotherapy is ideally started within eight weeks of surgery, and completion by ≤104 days is recommended [116]. 

For AdCC-BG, several series suggest improved survival with adjuvant RT after positive margins [76,79,130]. Other reports note relative radioresistance and suggest that high-LET radiotherapy (particularly carbon-ion RT) may provide superior local control compared with photons [73].

Special considerations for young, childbearing patients should be weighed [72]. Salvage stereotactic ablative radiotherapy to isolated perineural recurrence has also been reported with disease-free status at one year [131].

### 7.4. Chemotherapy Alone

Neoadjuvant chemotherapy for locally advanced BGC is not standard care but may be considered in selected patients who are not candidates for upfront surgery or primary chemoradiation, including for cytoreduction [116,132]. A case of locally advanced AdCC-BG responded radiologically to two cycles of cisplatin/paclitaxel before surgery [121]. In general, neoadjuvant chemotherapy is rarely used, and most recommendations are extrapolated from vulvar cancer. Adjuvant chemotherapy alone is not routinely recommended [35,133]. 

Systemic chemotherapy is typically reserved for palliation in symptomatic metastases or progressing disease [13].

### 7.5. Combined Therapy—Chemoradiation

Neoadjuvant chemoradiation may be used in locally advanced BGC with deep rectal and/or urethral infiltration to downstage disease, aiming to enable function-preserving surgery or provide symptomatic relief [4]. After neoadjuvant chemoradiation, radical vulvectomy has been reported with durable control in individual cases [134]. When used, fields usually include the vulva, groins, and pelvis. Cisplatin-based regimens are commonly employed (cisplatin plus paclitaxel, or cisplatin plus 5-fluorouracil) [2,61,122,135]. Case reports describe stable disease with cyclophosphamide/ doxorubicin/cisplatin or doxorubicin/ cisplatin for lung-metastatic AdCC-BG [136,137]. Postoperative adjuvant chemoradiation with irinotecan has also been described [138]. Neoadjuvant chemotherapy typically consists of 3–4 cycles, followed by restaging [116].

Chemoradiation can be used as definitive primary therapy in unresectable disease [116]. Primary chemoradiation has yielded long-term disease-free survival in basaloid SCC-BG infiltrating the anal canal [61]. In a cohort of 10 mixed-histology BGC patients treated with primary RT or chemoradiation, 3- and 5-year survivals were 71.5% and 66%, respectively, with outcomes similar to surgery plus adjuvant RT, suggesting that primary RT/CRT may be a function-preserving alternative with fewer complications [122]. Adjuvant chemoradiation is an option in node-positive disease; in a large vulvar-cancer analysis (*n* = 2770), chemoradiation improved 5-year survival vs. RT alone, particularly with ≥2 positive nodes [133]. GROINSS-V III supports consideration of definitive groin irradiation with concurrent cisplatin for macrometastatic SLN disease as an alternative to dissection in carefully selected early-stage cases [120]. Analogous strategies may be considered in BGC. Adjuvant chemoradiation has been recommended in the presence of vascular tumor emboli, positive margins, or groin nodal metastases [14].

### 7.6. Targeted Therapies

Targeted therapy may be used as second-line treatment after chemoradiation failure in metastatic or recurrent disease. Immune checkpoint inhibitors can be considered in SCC-BG; in KEYNOTE-158, pembrolizumab produced durable responses in advanced vulvar SCC regardless of PD-L1 status [115], and additional reports support a role in metastatic/recurrent SCC-BG [139,140]. The CheckMate 358 trial showed activity of nivolumab in metastatic/recurrent HPV-associated cervical, vaginal, and vulvar cancers [141]. For AdCC-BG, lenvatinib was used after lung relapse in a tumor with MYB-NFIB fusion and PLCG1 mutation [102]. In progressive (non-BG) AdCC, dovitinib achieved partial responses in 6% and stable disease ≥4 months in 65% [142]. BET inhibitors and the HDAC inhibitor romidepsin remain investigational but may have relevance given reported KDM6A alterations in salivary AdCC [112]. For BG adenocarcinoma, PTEN loss and CCND1 amplification may support mTOR inhibition and CDK4/6 blockade, respectively [106]. Bevacizumab with chemotherapy may be considered in HPV-related disease, though prospective second-line data in vulvar/BG cancer are lacking [116].

### 7.7. Treatment of Metastatic/Recurrent Disease

Management is multidisciplinary and tailored to performance status, disease burden (oligometastatic vs. disseminated), and prior therapy. Surgery for isolated local or groin recurrences has been proposed by some, noting limited benefit from chemotherapy and frequent contraindications to further RT; others outline options extrapolated from vulvar cancer—additional surgery, RT with or without chemotherapy, neoadjuvant chemotherapy with tailored therapy, palliative chemoradiation, or immunotherapy—which may be considered analogously in BGC [3,35]. Surgery may be appropriate for oligometastatic disease in fit patients; disseminated disease is usually managed with systemic chemotherapy and/or targeted agents. 

### 7.8. Follow-Up

Follow-up mirrors vulvar cancer: every six months for two years, then annually to five years; recurrence risk is highest during the first two years [35]. 

## 8. Conclusions

BGC is a rare malignancy for which high-level evidence is lacking; consequently, most treatment recommendations are extrapolated from vulvar cancer. The frequent clinical mimicry of cyst/abscess at initial presentation argues for a low threshold to biopsy solid, persistent, or recurrent “Bartholin” masses in women ≥40–45 years, to prevent stage migration from diagnostic delay. Histological subtype matters, as prognosis and management differ across SCC, AdCC, and adenocarcinoma. Molecular testing provides an essential aid for diagnosis and prognostication. A BGC registry with a harmonized minimal dataset (clinical presentation, imaging, histotype, HPV/p16/p53 status, nodal evaluation, treatment, margins, adjuvant therapy, patterns of failure, patient-reported outcomes), a centralized digital pathology review (including mandatory notation of HPV/p16/p53 and AdCC grading) and molecular re-review (targeted DNA panels with CNV, RNA fusion assays, and prespecified tissue-agnostic biomarkers: MMR/MSI, TMB, PD-L1) would reduce misclassification and identify trial eligibility. Based on molecular profiling, novel targeted agents and immunotherapies could contribute to individual treatment plans. BGC management in multidisciplinary oncological centers with molecular tumor boards is strongly advisable.

## Figures and Tables

**Table 1:** Histotype-specific molecular features in BGC.

Histotype	High-Value Molecular/ Biomarker Features	Practical Ancillary Tests	Diagnostic Relevance	Key References
SCC HPV-associated	HPV16/18 association; p16 block-positive	p16 IHC; HPV RNA/DNA ISH	Confirms HPV-driven pathway; segregates from HPV-independent SCC	[28,57]
SCC HPV-independent	Aberrant p53; TP53/CDKN2A; PIK3CA/HRAS; TERT-promoter; frequent CCND1 gains (± cyclin D1 overexpression); occasional EGFR amp; MMR-proficient (extrapolated)	p53 IHC; cyclin D1 IHC (CCND1 surrogate); broad NGS incl. CNV; EGFR FISH if relevant; PD-L1 IHC; eventually MMR/MSI/TMB (usually proficient/low)	Defines HPV-independent biology; CCND1/cyclin D1 refines prognosis (extrapolated)	[94,95,96,97,98,99,100]
AdCC-BG	MYB::NFIB or MYBL1 rearrangements; otherwise “quiet” genomes; rare AKT1/KDM6A/GNAS/GNAQ (case-level)	MYB IHC; MYB/MYBL1 FISH or RNA-seq	Fusion detection is diagnostic; not HPV-related.	[78,101,102]
Adenocarcinoma, intestinal-type	CK20+/CDX2+, variable CK7; SATB2 frequent; HPV-negative; KRAS/TP53 in a subset (extrapolated)	CK7/CK20/CDX2/SATB2 ± PAX8; GI work-up	Overlap with colorectal/anal primaries; metastases should be excluded.	[103,104,105]
Adenocarcinoma, non-intestinal	Case-level: PTEN loss (exons 2–5), CCND1 amplification by WGS	Broad NGS; to be discussed at molecular tumor board	Supports compassionate mTORi → CDK4/6i sequencing in selected cases	[106]

Abbreviations: AdCC = adenoid cystic carcinoma; AdCC-BG = adenoid cystic carcinoma of the BG; SCC = squamous cell carcinoma; SCC-BG = squamous cell carcinoma of the BG; VSCC = vulvar squamous cell carcinoma; HPV = human papillomavirus; IHC = immunohistochemistry; ISH = in situ hybridization; NGS = next-generation sequencing; RNA-seq = RNA sequencing; WGS = whole-genome sequencing; EGFR = epidermal growth factor receptor; FISH = fluorescence in situ hybridization; CK7/CK20 = cytokeratin 7/20; CDX2 = caudal type homeobox 2; SATB2 = special AT-rich sequence-binding protein 2; PAX8 = paired box 8; GI = gastrointestinal; TERT promoter = telomerase reverse transcriptase promoter.

**Table 2:** Key molecular biomarkers in BGC.

Biomarker/Pathway	Diagnostic/Biological Rationale	Preferred Method/ Platform	Potential Therapy/Management Impact	Evidence in BGC (vs. Extrapolated)	Key Refs
**HPV status (SCC-BG)**	Confirms HPV-driven pathway, aligns SCC-BG with HPV-related VSCC, explains p16 overexpression, younger age, better stage profile.	p16 IHC ± HPV RNA/DNA ISH	No de-escalation data in BGC, but HPV-positive tumors are the ones most analogous to ‘good-prognosis’ VSCC and to immunotherapy series in vulvar SCC.	**Direct** BG data (small series)	[28,57,99]
**p53 IHC pattern**	Separates HPV-independent squamous tumors from HPV-related ones; in VSCC, aberrant p53 is associated with worse outcome. The current three-tier classification system used in VSCC is applicable by analogy to SCC-BG.	p53 IHC, pattern-based interpretation	Frames prognosis in HPV-negative SCC-BG and tells you which tumors deserve broader sequencing.	**Extrapolated** from VSCC molecular subclassification	[94,96,100,110,111]
**Integrated HPV/p16 + p53 status**	Three prognostic VSCC groups (HPV+/p16+, HPV–/p53abn, HPV–/p53wt) have been defined in molecular subclassification; this framework can be applied analogously to SCC-BG.	p16 IHC + p53 IHC (both mandatory)	Helps reporting and follow-up stratification; still not a BGC-specific de-/escalation tool.	**Extrapolated** from VSCC	[95,96,97,98,105,106]
**CCND1 gain/Cyclin D1 overexpression**	CCND1/Cyclin D1 is a bad-risk signal in HPV-independent disease. Same biology is expected in HPV-independent SCC-BG.	Cyclin D1 IHC (screen) ± copy-number from targeted DNA NGS	Risk contextualization; in advanced/recurrent setting, could support considering CDK4/6 inhibitor concepts.	**Extrapolated** from VSCC	[97,98,100]
**MYB/MYBL1 rearrangements (AdCC-BG)**	Near-pathognomonic for BG adenoid cystic carcinoma; absence should trigger re-evaluation.	MYB IHC → FISH or RNA-based fusion panel	Diagnostic confirmation; occasionally eligibility for ACC-type trials.	**Direct** BG AdCC case series/reports	[78,102,113]
**PI3K–mTOR/cell-cycle lesions in AC-BG (e.g., PTEN loss, CCND1 amp)**	Whole-genome BG adenocarcinoma with PTEN loss + CCND1 amp that responded to everolimus then palbociclib.	Broad DNA NGS with CNV calling; RNA optional	Supports off-label/compassionate use (mTORi → CDK4/6i sequence).	**Direct** but single-patient	[99,106]
**EGFR amplification/9p24 gains (SCC-BG)**	Seen in VSCC WES; marks a more aggressive HPV-negative subset.	DNA NGS with CNV or FISH	Currently only for trial or n-of-1 decisions; no BG-specific response data.	**Extrapolated** from VSCC	[98,100]
**MMR/MSI, TMB-H, PD-L1**	Tissue-agnostic biomarkers; PD-L1 positivity is more common in HPV-negative VSCC, so likely also in HPV-negative SCC-BG.	IHC for MMR and PD-L1; NGS for MSI/TMB	Supports use of pembrolizumab or other checkpoint inhibitors as in KEYNOTE-158 vulvar SCC cohort.	**Extrapolated** from vulvar SCC trials	[115]
**KRAS (intestinal-type AC-BG)**	Confirms intestinal-type differentiation and supports exclusion of colorectal origin. KRAS p.G12D has been directly demonstrated in a true BG adenocarcinoma case.	Targeted DNA NGS (KRAS exon 2–4)	Argues against EGFR-targeted therapy; supports GI-style work-up when metastasis is suspected.	**Direct** single-case + **extrapolated** vulvar intestinal-type series	[68,70,105]
**‘Broad panel’/global actionability**	VSCC WES shows virtually every tumor has ≥1 potentially targetable alteration or immune biomarker; reasonable to expect the same or higher in advanced BGC.	Comprehensive hybrid-capture DNA ± RNA NGS	Opens trial eligibility; informs compassionate treatment in recurrence.	**Extrapolated** from VSCC WES	[97,99,100,109,110]

Abbreviations: AC-BG = adenocarcinoma of the BG; AdCC-BG = adenoid cystic carcinoma of the BG; SCC-BG = squamous cell carcinoma of the BG; MMR = mismatch repair; MSI = microsatellite instability; TMB(-H) = tumor mutational burden (high); PD-L1 = programmed death-ligand 1; mTORi = mTOR inhibitor; CDK4/6i = CDK4/6 inhibitor; KRAS p.G12D = Gly→Asp substitution at codon 12 (activating mutation); plus all abbreviations from Table 1.

**Table 3:** Levels and sources of evidence for treatment modalities in VSCC and BGC.

Treatment Modality	VSCC	BGC
1. Surgical treatment of the primary tumor	L 3–4, [116]	L 4–5, direct [1,2,3]
2. Groin treatment	L 3–4, [116]	L 4–5, direct [1,2,3,35,64,117,118]
3. Sentinel lymph node procedure	L 1–2, [35,116]	V, indirect, [35,116]
4. Adjuvant radiotherapy to the vulva	L-4, [35,116]	L-4, direct, [5,6,119]
5. Adjuvant radiotherapy to the groin	L 2–3, [35,116]	L-4, direct, [5,6,119]
6. Adjuvant chemoradiotherapy	L-3, [116,120]	L-4, direct, [5,6,7]
7. Neoadjuvant chemotherapy	L-4, [116]	L-5, direct, [121]
8. Neoadjuvant chemoradiotherapy	L-3, [35,116]	L-4, direct, [122]
9. Targeted therapies	L 3–4, [35,116]	L-5, direct, [102,106]
10. Recurrent/metastatic disease [systemic therapy]	L 3–4, [35,116]	L-5, indirect, [35,116]

Level of evidence: L-1: evidence from at least one large randomized controlled trial or meta-analysis; L-2: small randomized trials or large randomized trials with suspected bias; L-3: prospective cohort data; L-4: retrospective studies or case series; L-5: studies without a control group, case reports, or expert opinion. Source of evidence (for BGC): *Direct* = BGC-specific data; *indirect* = extrapolated from VSCC/vulvar cancer cohorts and guidelines.

## Appendix A

**Table A1:** Levels and sources of evidence for preoperative diagnostic modalities in BGC and VSCC.

Preoperative Diagnostic Modality	VSCC	BGC
Biopsy of the primary tumor	Small biopsy of all suspicious areas—L-5, [116]	Formal en bloc excision—L-5, direct, [4]
Evaluation of cervix, vagina and anus, including cytology and HPV test from cervix/vagina	L-4, [116]	L-4, direct [3,5,28]
Imaging for pT1a tumors not required	L-3, [116]	L-5, indirect [116]
Imaging for all other stages [expert vulvar sonography, CT or PET/CT]	L-3, [116]	L-5, indirect [116]
Lymph node status–ultrasound-guided fine-needle aspiration or core needle biopsy	L-3 [6,37,116]	L-5 indirect [36,37,102]
MRI in locally advanced tumor with involvement of surrounding tissue	L-4 [116]	L-4, direct [2,3,5]
Biopsy of distant metastases	L-5 [116]	L-5, indirect [116]

Level of evidence: L-1: evidence from at least one large randomized controlled trial or meta-analysis; L-2: small randomized trials or large randomized trials with suspected bias; L-3: prospective cohort data; L-4: retrospective studies or case series; L-5: studies without a control group, case reports, or expert opinion. Source of evidence (for BGC): *Direct* = BGC-specific data; *indirect* = extrapolated from VSCC/vulvar cancer cohorts and guidelines. **Abbreviations:** VSCC, vulvar squamous cell carcinoma; BGC, Bartholin gland carcinoma; CT, computed tomography; PET/CT, positron emission tomography/computed tomography; MRI, magnetic resonance imaging.
